# Operation of Lithotomy

**Published:** 1846-07

**Authors:** 


*Operation of Lithotomy.*—We perceive by the Jeffersonian, published at Watertown, N. Y., that the veteran Surgeon, Prof. Trowbridge, of that village, has recently operated with success for Lithotomy. The case appears to have been one of unusual interest, from the fact that the patient for many years had resorted to different systems of quackery, and a succession of patent medicines, the real difficulty not having been suspected. We must add, that we regret to see this, or any other case made the subject of a newspaper report, or rather bulletin. Some admirer of the worthy Professor, doubtless permitted his zeal to outrun his discretion. Prof. T's reputation as an operative Surgeon does not require any bolstering of that kind, and we are sure that he would be unwilling to sanction it.

